# Impact of Socio-Economic Factors and Health Information Sources on Place of Birth in Sindh Province, Pakistan: A Secondary Analysis of Cross-Sectional Survey Data

**DOI:** 10.3390/ijerph16060932

**Published:** 2019-03-15

**Authors:** Jin-Won Noh, Young-mi Kim, Nabeel Akram, Ki-Bong Yoo, Jooyoung Cheon, Lena J. Lee, Young Dae Kwon, Jelle Stekelenburg

**Affiliations:** 1 Department of Healthcare Management, Eulji University, Seongnam 13135, Korea; jw.noh@eulji.ac.kr; 2 Global Health Unit, Department of Health Sciences, University Medical Centre Groningen, University of Groningen, Groningen 9713 GZ, The Netherlands; jelle.stekelenburg@online.nl; 3 Jhpiego, Johns Hopkins University, Baltimore, MD 21231, USA; Young-Mi.Kim@jhpiego.org (Y.-M.K.); nabeel.akram@jhpiego.org (N.A.); 4 Department of Health Administration, Department of Information & Statistics, Yonsei University, Wonju 26493, Korea; ykbong@yonsei.ac.kr; 5 Department of Nursing Science, Sungshin University, Seoul 01133, Korea; jcheon@sungshin.ac.kr; 6 National Institutes of Health Clinical Center, Bethesda, MD 20892, USA; jumin.park@nih.gov; 7 Department of Humanities and Social Medicine, College of Medicine and Catholic Institute for Healthcare Management, The Catholic University of Korea, Seoul 06591, Korea; 8 Department of Obstetrics and Gynecology, Medical Centre Leeuwarden, Leeuwarden 8934 AD, The Netherlands

**Keywords:** maternal-child health, socio-economic factor, health information source, place of birth, Pakistan

## Abstract

Medical facility birth with skilled birth attendance is essential to reduce maternal mortality. The purpose of this study was to assess the demographic characteristics, socio-economic factors, and varied health information sources that may influence the uptake of birth services in Pakistan. We used pooled data from Maternal-Child Health Program Indicator Survey 2013 and 2014. Study population was 9719 women. Generalized linear model with log link and a Poisson distribution was used to identify factors associated with place of birth. 3403 (35%) women gave birth at home, and 6316 (65%) women gave birth at a medical facility. After controlling for all covariates, women’s age, number of children, education, wealth, and mother and child health information source (doctors and nurses/midwives) were associated with facility births. Women were significantly less likely to give birth at a medical facility if they received maternal-child health information from low-level health workers or relatives/friends. The findings suggest that interventions should target disadvantaged and vulnerable groups of women after considering rural-urban differences. Training non-health professionals may help improve facility birth. Further research is needed to examine the effect of individual information sources on facility birth, both in urban and rural areas in Pakistan.

## 1. Introduction

Maternal mortality is one of the greatest health and development concerns worldwide, especially in the developing world [1]. Over the course of the Millennium Development Goals (1990–2015), the global maternal mortality ratio (MMR) declined substantially. However, many low-income countries did not reach the target for Millennium Development Goal 5, which aimed for a 75% reduction in the MMR between 1990 and 2015 [2,3]. Compared with other low- and middle-income countries, the MMR in Pakistan remains high and the decline has been extremely slow [4,5]. Furthermore, the MMR in Pakistan is almost twice as high in rural as urban areas (319 versus 175 deaths per 100,000 live births) [6]. 

Facility birth can make a difference. Appropriate medical attention and hygienic conditions during birth decrease the risk of complications and infections that may lead to death or serious illness for the mother, the baby, or both. Therefore, increasing the proportion of babies delivered in a safe and clean environment under the supervision of qualified and experienced health professionals is important for the survival and well-being of the mother and her child [7,8,9]. In addition, skilled birth attendance has a positive impact on childhood immunization coverage, ultimately increasing child survival [10].

Pakistan’s National Maternal, Newborn and Child Health Program has introduced trained community midwives in rural areas and provided birth services by lady health visitors in basic health units and rural health centers to increase skilled birth attendance. However, more than half of births (52%) occurred at home without skilled attendance, according to the 2012–2013 Pakistan Demographic and Health Survey (PDHS) [8]. Hence it is vital to identify the barriers and facilitators affecting women’s decisions about whether to give birth at home or at a medical facility. Understanding these determinants is important to develop appropriate interventions, health systems, and health policies to improve the use of birth care services and ultimately achieve the reduction in MMR [7,11,12,13]. 

Previous studies have identified several socioeconomic factors associated with the use of a medical facility birth, including mother’s education, husband’s education, household wealth, and residence [8,11,12,14,15,16,17]. Information sources on maternal-child health (MCH) also may influence facility deliveries by increasing women’s knowledge regarding the importance of giving birth at a facility, although this has been less well studied than socio-economic status and results are controversial. Some studies have found that lack of exposure to health information in the mass media decreases the likelihood of women delivering at a medical facility [16,17]. In contrast, another study [14] found no significant association between receiving MCH information from a Lady Health Worker (LHW) and medical facility birth.

In-depth investigations of the determinants of women’s decisions regarding home versus facility deliveries in Pakistan, especially socio-economic factors and information sources, are limited. The purpose of this study was to assess the demographic characteristics, socio-economic factors, and varied health information sources that may affect the uptake of birth services in Pakistan.

## 2. Methods

### 2.1. Sampling and Subjects

This study used a subset of data drawn from two rounds of a larger household survey conducted in Sindh Province, Pakistan, with the support of the United States Agency for International Development (USAID). The MCH Program Indicator Survey 2013 and 2014 was a cross-sectional survey that employed a multi-stage stratified sampling design using district-level population information [18]; districts are the third-order administrative division of Pakistan. The sampling process was designed to yield a representative sample at the provincial level. Trained interviewers visited each selected household. Participants were limited to women aged 15–49 years who had a live birth in the two years before the survey. One eligible woman per household was randomly selected. As female literacy is low in Sindh Province, female interviewers obtained informed consent verbally from each respondent and then signed the consent form on behalf of the respondent. The overall refusal rate was 15%. The analysis included 10,200 respondents (4000 from the 2013 survey and 6200 from the 2014 survey); 481 respondents were excluded because of missing data, resulting in a study group of 9719. Of these, 35% (3403 women) gave birth at home, and 65% (6316 women) gave birth at a medical facility. The sample size of this study was calculated based on the article of Peduzzi et al. (1996) and the Medcalc manual [19]. The suggested minimum number of samples is approximately 371. The number of samples in each subgroup is over 2000. So the sample size is enough to conduct the regression.

The study was approved by the Johns Hopkins University School of Public Health Institutional Review Board (IRB #00005002) and the National Bioethics Committee of Pakistan. Both Institutional Review Boards approved the verbal consent. Married women 15 and older are considered adults in the cultural context of Pakistan. Many of these women may live in nuclear households where no elder relative is in co-residence. National surveys conducted in Pakistan, including the Demographic and Health Surveys conducted in 2006–2007 and in 2011–2012, regularly interview married women 15 and older. They also collect data from married women 15 and older (15–49 to be precise) in order for this survey to be comparable to other surveys which have been conducted in Pakistan. They requested Institutional Review Boards permission to conduct interviews with married women 15 and older without seeking permission from an older adult or guardian. Female interviewers obtained informed consent verbally from each respondent and then signed the consent form on behalf of the respondent. Interviewers were required to sign the consent form attached to the survey questionnaire to confirm that they had read the informed consent script.

### 2.2. Variables

Place of birth was the dependent variable. Interviewers asked “Where did you give birth?” (referring to women’s last live birth), and responses were categorized into home (own home, other home) or medical facility (private hospital/clinic, government hospital, rural health centers, MCH centers). 

Demographic characteristics, socio-economic characteristics, and MCH information sources were the independent variables. Demographic characteristics included women’s age (15–24, 25–34, 35) and number of living children (1, 2, 3, 4). Socio-economic characteristics included residence (rural, town or small city, large city), women’s education (no education, primary or middle, secondary or higher), husband’s education level (no education, primary or middle, secondary or higher), and household wealth (quintiles). The wealth index was derived from the household ownership of assets by principal components analysis and classified into quintiles [8].

For MCH information sources, interviewers asked, “During the last 12 months have you received any information about MCH from the following sources?” Respondents could select multiple responses. Responses were categorized into: doctor, nurse/midwife, lady health visitor, low-level health workers (Dai-traditional birth attendant, LHW, homeopath, Hakim herbal medicine practitioner, outreach worker), relatives/friends (mother-in-law, other relative, friend), and media.

### 2.3. Statistical Analysis

The data from the 2013 and 2014 surveys were pooled. Proportion between the categorical variables was tested by Chi-square test. Then generalized linear model with log link and a Poisson distribution was used to identify the factors associated with place of birth. Due to high rate of facility birth, log-binomial regression is better than logistic regression. Because of fail convergence, we decided to use that model including robust variance estimates [20]. Residence was a potential confounding factor, because of differences in availability of and access to medical facilities [21], inequities in antenatal care services, and social development ranking [22]. Therefore, we conducted a subgroup analysis for residence. A priori, based on existing evidences [8,11,12,14,15,16,17], demographic factors (women’s age, number of living children), socioeconomic factors (residence, woman’s education, husband’s education, household wealth), and MCH information sources (doctor, nurse/midwife, lady health visitor, relatives/friends, media) were identified as likely predictors and included in the model. A *p*-value of 0.05 was used to determine statistical significance. All statistical analyses were performed using SAS software (SAS Institute, Inc., Cary, NC, USA) version 9.4.

## 3. Results

Most women were age 25–34 (*n* = 5444, 56.0%), had four or more children (*n* = 3696, 38.0%), and had no education (*n* = 5818, 59.9%) (Table 1).

Almost half (*n* = 4969, 51.1%) lived in rural areas. The likelihood of a facility birth significantly decreased with women’s age and number of children. It significantly increased with women’s education, husband’s education, and wealth. Residence was also associated with place of birth: rural women were most likely to give birth at home, while women in large cities were most likely to give birth at a medical facility. Facility birth was positively associated with receiving MCH information from all but one information source: low-level health workers (Table 1). The results of generalized linear model with log link and a Poisson distribution are shown in Table 2. 

They confirm the link between facility birth and women’s age, number of children, education, and wealth. After controlling for all other variables, wealth was the strongest determinant: women in the top wealth quintile were more likely to have a facility birth than women in the bottom quintile (relative risk (RR) = 1.84; 95% confidence interval (CI): 1.71–1.98). Women’s education (RR = 1.19 for women with secondary or higher education; 95% 1.14–1.23) was a stronger determinant of facility birth than husband’s education (RR = 1.09 for husbands with secondary or higher education; 95% CI: 1.05–1.13). 

The impact of MCH information sources was mixed. Women were significantly more likely to give birth at a medical facility if they received MCH information from doctors (RR = 1.23; 95% CI: 1.20–1.27) or nurses/midwives (RR = 1.09; 95% CI: 1.05–1.13). However, women were significantly less likely to give birth at a medical facility if they received MCH information from low-level health workers (RR = 0.89; 95% CI: 0.85–0.94) or relatives/friends (RR = 0.92; 95% CI: 0.89–0.95). There was no association with information received from lady health visitors or the media (Table 2).

Residence itself was not a significant determinant of facility birth in the result. However, the subgroup analysis in Table 3 shows that other determinants of facility birth varied by women’s residence. The number of children was significant everywhere. While women’s education was significant everywhere, husband’s education was significant in rural areas and large cities. Wealth was the strongest determinant of all in rural areas (RR = 1.92; 95% CI: 1.74–2.12) and in towns/small cities (RR = 1.63; 95% CI: 1.30–2.03), but was not significant in large cities. Doctors as source of health information were positively associated with facility birth in all residence groups, whilst this association was significant in rural areas (RR = 1.19; 95% CI: 1.11–1.29) and in large cities (RR = 1.04; 95% CI: 1.00–1.09) for nurses as source of information. Otherwise, lady health visitors as source of health information were not significantly associated with facility birth in all subgroups, whilst this association was negatively significant in rural areas (RR = 0.87; 95% CI: 0.80–0.94) and towns/small cities (RR = 0.90; 95% CI: 0.82–0.98) for low-level health workers and in rural areas (RR = 0.86; 95% CI: 0.81–0.91) and large cities (RR = 0.96; 95% CI: 0.92–0.99) for relatives/friends as source of information (Table 3).

## 4. Discussion

Low rates of facility birth pose a continuing challenge for Pakistan’s efforts to reduce maternal mortality. In this study we aimed to identify the factors—demographic, socio-economic and information sources - that influence women’s choices about where to give birth in Sindh Province. The facility birth rate in this study was 65% overall, but it was 1.5 times higher in large cities (84%) than in rural areas (53%). This is consistent with previous findings showing that the proportion of women giving birth at health facilities was 1.5 to 4 times higher in urban than rural areas of Pakistan [8,23,24]. Women living in large cities have easier access to and more opportunities to use facility-based maternal health services because of their higher socio-economic status, the greater density of skilled health professionals (especially female doctors), and better transportation, communication, and other infrastructure. Urban advantages and rural disadvantages may result from differential government investments, remoteness of the location, lack of necessity to give birth in the medical facilities, and cultural differences [14,25,26,27]. 

The best educated women were more likely to give birth in a medical facility than the least educated, even after controlling for all other factors. The fact that educated women are more likely to delay their first birth until a later age, as other studies have found, may contribute to their use of health facilities for giving birth [8,24,28,29,30]. In addition, educated mothers may attach greater importance to their health and may be more aware of potential risks during birth. It is also possible that more educated mothers have greater autonomy to make healthcare decisions, greater confidence in their healthcare providers’ ability, and greater willingness to travel outside their homes [28,30]. 

Interestingly, husband’s educational level was also a significant predictor of facility birth in rural areas and in large cities. In traditional Muslim culture, where women are not allowed to step out alone without their husband’s approval, men play a paramount role in deciding when and where women should seek health care, and their interest or approval is an important determinant of use of health services of all kinds [28,29,31]. Half of Pakistani women have no power to make decisions about their own health care and medical purchases [8,27,28]. Given men’s role in family life, raising husbands’ awareness of potential birth complications, treatment options, and the importance of facility birth could be important for Pakistani women [28,29]. Especially, distance to the medical facility in rural areas [8] may exacerbate the situation, because husbands must take women to the facility. Furthermore, cultural preference for home birth, distrust of medical facilities, or husbands’ disinterest in the health of their wives may be also barriers to facility birth.

In this study, household wealth was an important determinant of facility birth in rural areas, towns and small cities, but not in large cities. This supports previous findings that poor women choose to give birth at home because it is a cheaper option, especially in rural areas where medical facilities are more distant and transportation costs are higher [12,24,27,28,30]. In contrast, women living in big cities have good health infrastructure and easy access to facilities, so household wealth has less impact on health care utilization, including facility birth [14,25,32,33].

Notably, the impact of receiving MCH information on facility birth varied dramatically in this study, depending on the source of that information. Information from skilled birth attendants, including doctors, nurses, and midwifes, encouraged facility birth. However, information from lady health visitors, low-level health workers, and relatives and friends either had no impact or, in some locations, discouraged facility deliveries—suggesting that these information sources are not reinforcing, and may even be undermining, government messages promoting facility birth. Some lady health visitors assist at home deliveries in Pakistan [32,34] as do traditional birth attendants and lady health workers [32], so they may have a vested interest in the continuation of home deliveries. Conservative Muslim family members, especially husbands, mothers-in-law, and grandmothers, have a cultural preference for home deliveries and may prohibit pregnant women from going to a hospital to give birth [29,31]. 

In this study, the media was not an influential information source for facility birth. A study using 2013 survey data found that the most important source of information regarding place of birth in Sindh was doctors (21%); only 14% of women said television was an important information source [18]. Another study found that mass media exposure was associated with facility birth in rural areas of Pakistan [28], but it did not adjust for other health information sources, such as doctors and nurses, unlike our study. Further research is needed to examine the effect of individual information sources on facility birth after adjusting for demographic and socioeconomic determinants in urban and rural areas in Pakistan.

Policy makers and program managers in Sindh Province can use the results of this study to devise more effective strategies to promote facility birth and decrease MMR. In rural areas, interventions should target women who are uneducated, poor, and having no information source from doctors, nurses and midwives. The effect of a low-cost voucher scheme and reimbursement of travel costs on facility birth was proven in a single rural area in Pakistan [28], therefore, provision of low-cost/free vouchers and incentives for travel could help improve facility birth and could be as part of interventions for facility birth in Pakistan. The lady health visitors and low-level health workers should be trained to motivate Pakistani women to use maternal health care services by emphasizing the benefits of facility birth and should act as a mediator between Pakistani women and medical facilities [25]. Establishing a well-organized referral system for lady health visitors and low-level health workers shall help Pakistani women’s transition from home birth to facility birth. 

Encouraging more women to make use of institutional maternal health services obliges policy maker and health workers to ensure adequate quality of antenatal care and emergency obstetric care. Too frequently women are encouraged to give birth in health institutions where quality of care is not guaranteed, which could even pose more risk on the health of mothers and babies than giving birth at home. Timely access to quality maternal health services is the key to decreasing maternal and neonatal complications.

This study has limitations. As noted, the data come from one single province so the findings cannot be generalized to all of Pakistan. Second, this study did not control for some important determinants of the utilization of maternal health service, such as complications during pregnancy and birth, past use of healthcare services, and distance from health facilities. Third, the variable regarding MCH information sources may not adequately represents whether information sources within the past 12 months had an impact on women’s choice of delivery location because the study participants were women who had a live birth in the two years before the survey. Lastly, this cross-sectional study could not make causal inferences from the findings. Despite the limitations, the strength of the present study was the use of large sample of Pakistani women in Sindh using recent data from MCH Program Indicator Survey. The study findings suggested the evidence for target populations to improve facility birth by identifying socially disadvantaged and vulnerable groups, and for the need of training program among non-health professionals.

## 5. Conclusions

The study findings suggest that interventions to promote facility birth in Pakistan should target young women with lower socio-economic status, especially poor and uneducated, and women who live in rural areas with uneducated husbands, and who receive MCH information from non-health professionals. Educating women and their husbands to use health facilities for varied services and training lady health visitors and low-level health workers may help encourage women to give birth there. Policy makers should focus on rural-urban differences, identification of disadvantaged and vulnerable women, the density and composition of the health work force, and recognize and address cultural and religious factors, which may restrict maternal health-seeking behavior.

## Figures and Tables

**Table 1:** Characteristics of respondents, by place of birth.

Characteristic		Birth at				Total (*n* = 9719)	*p*-Value
		Home (*n* = 3403)		Medical Facility (*n* = 6316)			
		*n*	%	*n*	%	*n*	
Demographic characteristics							
Women’s age	15–24	923	31.6	1997	68.4	2920	<0.001
	25–34	1921	35.3	3523	64.7	5444	
	35+	559	41.3	796	58.7	1355	
Number of living children	1	520	23.6	1679	76.4	2199	<0.001
	2	673	30.8	1515	69.2	2188	
	3	559	34.2	1077	65.8	1636	
	4+	1651	44.7	2045	55.3	3696	
Socio-economic characteristics							
Residence	Rural	2205	47.0	2491	53.0	4696	<0.001
	Town/Small city	804	31.0	1791	69.0	2595	
	Large city	394	16.2	2034	83.8	2428	
Woman’s education	No education	2750	47.3	3068	52.7	5818	<0.001
	Primary or middle	477	24.0	1512	76.0	1989	
	Secondary or higher	176	9.2	1736	90.8	1912	
Husband’s education	No education	1811	48.3	1936	51.7	3747	<0.001
	Primary or middle	752	34.7	1414	65.3	2166	
	Secondary or higher	840	22.1	2966	77.9	3806	
Household wealth	First/poorest	1221	61.4	768	38.6	1989	<0.001
	Second	904	46.2	1053	53.8	1957	
	Third	677	34.8	1267	65.2	1944	
	Fourth	430	22.3	1499	77.7	1929	
	Fifth/richest	171	9.0	1729	91.0	1900	
Mother and child health information received from:							
Doctor	No	2436	43.5	3164	56.5	5600	<0.001
	Yes	967	23.5	3152	76.5	4119	
Nurse/Midwife	No	3159	36.7	5441	63.3	8600	<0.001
	Yes	244	21.8	875	78.2	1119	
Lady health visitor	No	3184	36.0	5665	64.0	8849	<0.001
	Yes	219	25.2	651	74.8	870	
Low-level health workers ^†^	No	2946	34.4	5626	65.6	8572	<0.001
	Yes	457	39.8	690	61.2	1147	
Relatives/friends	No	2039	38.9	3210	61.2	5249	<0.001
	Yes	1364	30.5	3106	69.5	4470	
Media	No	2933	39.0	4581	61.0	7514	<0.001
	Yes	470	21.3	1735	78.7	2205	
Survey Year	2013	1386	36.2	2438	63.8	3824	0.041
	2014	2017	34.2	3878	65.8	5895	

^†^ Low-level health workers include: Dai-traditional birth attendant, lady health worker, homeopath, Hakim-herbal medicine practitioner, outreach worker.

**Table 2:** Generalized linear model with log link and a Poisson distribution: medical facility birth by demographic and socio-economic characteristics and maternal and child health information sources.

Characteristic		Relative Risk (95% Confidence Interval (CI))
Demographic characteristics		
Women’s age	15–24	1.00
	25–34	1.03 (0.99–1.06)
	35+	1.08 (1.03–1.14) **
Number of living children	1	1.00
	2	0.91 (0.88–0.94) ***
	3	0.88 (0.85–0.92) ***
	4+	0.81 (0.79–0.84) ***
Socio-economic characteristics		
Residence	Rural	1.00
	Town/small city	0.97 (0.93–1.01)
	Large city	1.00 (0.96–1.04)
Woman’s education	No education	1.00
	Primary or middle	1.14 (1.10–1.18) ***
	Secondary or higher	1.19 (1.14–1.23) ***
Husband’s education	No education	1.00
	Primary or middle	1.07 (1.02–1.11) **
	Secondary or higher	1.09 (1.05–1.13) ***
Household wealth	First/poorest	1.00
	Second	1.35 (1.26–1.44) ***
	Third	1.55 (1.45–1.66) ***
	Fourth	1.71 (1.59–1.83) ***
	Fifth/richest	1.84 (1.71–1.98) ***
Mother and child health information received from:		
Doctor	No	1.00
	Yes	1.23 (1.20–1.27) ***
Nurse/midwife	No	1.00
	Yes	1.09 (1.05–1.13) ***
Lady health visitor	No	1.00
	Yes	1.01 (0.97–1.05)
Low–level health workers ^†^	No	1.00
	Yes	0.89 (0.85–0.94) ***
Relatives/friends	No	1.00
	Yes	0.92 (0.89–0.95) ***
Media	No	1.00
	Yes	1.00 (0.98–1.03)
Survey Year	2013	1.00
	2014	1.01 (0.98–1.04)

* *p* < 0.05; ** *p* < 0.01; *** *p* < 0.001. ^†^ Low-level health workers included Dai-traditional birth attendant, lady health worker, homeopath, Hakim-herbal medicine practitioner, outreach worker.

**Table 3:** Generalized linear model with log link and a Poisson distribution: results of residence subgroup analysis to choose a medical facility as the place of birth.

Characteristic		Rural (*n* = 4696)	Town/Small City (*n* = 2595)	Large City (*n* = 2428)
		RR (95% CI)	RR (95% CI)	RR (95% CI)
Demographic characteristics				
Women’s age	15–24	1.00	1.00	1.00
	25–34	1.03 (0.97–1.09)	1.06 (0.99–1.12)	1.01 (0.97–1.05)
	35+	1.07 (0.98–1.18)	1.09 (0.99–1.2)	1.08 (1.01–1.16) *
Number of living children	1	1.00	1.00	1.00
	2	0.83 (0.77–0.89) ***	0.93 (0.87–0.99) *	0.97 (0.93–1.00)
	3	0.82 (0.76–0.89) ***	0.88 (0.82–0.95) **	0.96 (0.92–1.00)
	4+	0.77 (0.71–0.83) ***	0.81 (0.76–0.88) ***	0.86 (0.81–0.91) ***
Socio-economic characteristics				
Woman’s education	No education	1.00	1.00	1.00
	Primary or middle	1.14 (1.07–1.21) ***	1.10 (1.03–1.17) **	1.20 (1.12–1.28) ***
	Secondary or higher	1.16 (1.07–1.25) ***	1.14 (1.06–1.21) ***	1.27 (1.20–1.35) ***
Husband’s education	No education	1.00	1.00	1.00
	Primary or middle	1.11 (1.04–1.19) **	1.00 (0.92–1.09)	1.07 (1.01–1.14) *
	Secondary or higher	1.13 (1.06–1.2) ***	1.06 (0.99–1.14)	1.06 (1.00–1.12) *
Wealth	First/poorest	1.00	1.00	1.00
	Second	1.31 (1.22–1.41) ***	1.28 (1.02–1.6) *	1.85 (0.33–10.32)
	Third	1.55 (1.43–1.67) ***	1.37 (1.10–1.70) **	1.81 (0.33–9.89)
	Fourth	1.76 (1.62–1.92) ***	1.50 (1.21–1.87) ***	2.07 (0.38–11.29)
	Fifth/richest	1.92 (1.74–2.12) ***	1.63 (1.30–2.03) ***	2.23 (0.41–12.16)
Mother and child health information received from:				
Doctor	No	1.00	1.00	1.00
	Yes	1.34 (1.27–1.42) ***	1.21 (1.14–1.28) ***	1.12 (1.07–1.16) ***
Nurse/Midwife	No	1.00	1.00	1.00
	Yes	1.19 (1.11–1.29) ***	1.03 (0.97–1.11)	1.04 (1.00–1.09) *
Lady health visitor	No	1.00	1.00	1.00
	Yes	0.99 (0.91–1.08)	1.05 (0.98–1.12)	0.95 (0.89–1.01)
Low-level health workers ^†^	No	1.00	1.00	1.00
	Yes	0.87 (0.80–0.94) **	0.90 (0.82–0.98) *	0.94 (0.86–1.02)
Relatives/friends	No	1.00	1.00	1.00
	Yes	0.86 (0.81–0.91) ***	0.99 (0.93–1.06)	0.96 (0.92–0.99) *
Media	No	1.00	1.00	1.00
	Yes	1.00 (0.94–1.07)	0.98 (0.93–1.04)	1.03 (0.99–1.07)
Survey Year	2013	1.00	1.00	1.00
	2014	1.06 (1.00–1.12) *	0.99 (0.93–1.04)	0.99 (0.95–1.02)

RR, relative risk; CI, confidence interval. * *p* < 0.05; ** *p* < 0.01; *** *p* < 0.001. ^†^ Low-level health workers included Dai-traditional birth attendant, lady health worker, homeopath, Hakim-herbal medicine practitioner, outreach worker.
